# Metformin Counteracts the Deleterious Effects of Methylglyoxal on Ovalbumin-Induced Airway Eosinophilic Inflammation and Remodeling

**DOI:** 10.3390/ijms24119549

**Published:** 2023-05-31

**Authors:** Matheus L. Medeiros, Akila L. Oliveira, Glaucia C. Mello, Edson Antunes

**Affiliations:** Department of Translational Medicine, Pharmacology Area, Faculty of Medical Sciences, University of Campinas (UNICAMP), Alexander Fleming St., Campinas 13083-881, SP, Brazil; matheuslmedeiros19@gmail.com (M.L.M.); akilalara.1@gmail.com (A.L.O.);

**Keywords:** mucus, collagen, RAGE, reactive-oxygen species, Th2-cytokines, eotaxin

## Abstract

Exposure to methylglyoxal (MGO) increases the levels of receptor for advanced glycation end products (RAGE) and reactive-oxygen species (ROS) in mouse airways, exacerbating the inflammatory responses. Metformin scavenges MGO in plasma of diabetic individuals. We investigated if amelioration by metformin of eosinophilic inflammation reflects its ability to inactivate MGO. Male mice received 0.5% MGO for 12 weeks together or not with 2-week treatment with metformin. Inflammatory and remodeling markers were evaluated in bronchoalveolar lavage fluid (BALF) and/or lung tissues of ovalbumin (OVA)-challenged mice. MGO intake elevated serum MGO levels and MGO immunostaining in airways, which were reduced by metformin. The infiltration of inflammatory cells and eosinophils and levels of IL-4, IL-5 and eotaxin significantly increased in BALF and/or lung sections of MGO-exposed mice, which were reversed by metformin. The increased mucus production and collagen deposition by MGO exposure were also significantly decreased by metformin. In MGO group, the increases of RAGE and ROS levels were fully counteracted by metformin. Superoxide anion (SOD) expression was enhanced by metformin. In conclusion, metformin counteracts OVA-induced airway eosinophilic inflammation and remodeling, and suppresses the RAGE-ROS activation. Metformin may be an option of adjuvant therapy to improve asthma in individuals with high levels of MGO.

## 1. Introduction

Asthma is a chronic inflammatory disease of the airways that represents a serious global health problem and affects many people with a greater severity in children [1]. Asthma symptoms comprising wheezing, coughing, chest tightness and shortness of breath are attributed to airflow obstruction and bronchial hyperresponsiveness [2,3]. T helper 2 lymphocytes (Th2) are the main producers of the pro-inflammatory cytokines interleukin (IL)-4, IL-5 and IL-13, as well as of the chemokine eotaxin, which, in combination, efficiently recruit eosinophils to lung tissue of asthmatic individual [4]. In the airways release, activated eosinophils release several preformed cytotoxic mediators including major basic protein (MBP), eosinophil cationic protein (ECP), eosinophil peroxidase (EPO) and eosinophil-derived neurotoxin (EDN), and newly synthesized inflammatory mediators like eicosanoids, platelet-activating factor (PAF), oxygen-reactive species, cytokines and neuropeptides [2]. There are multiple risk factors associated with asthma in children and adults, including exposures to allergen, viruses, tobacco smoke and air pollution; additionally, not neglecting the genetic basis of the individual plays a role in determining susceptibility to these factors [3,4]. Obesity is another important risk factor that increases asthma severity and reduces the efficacy of first-line medications to treat this respiratory disease [5]. Chronic high intake of fructose from sugar-sweetened beverages with added sugar like sodas, fruit drinks, sports/energy drinks, pre-sweetened iced tea and artificially sweetened homemade beverages has been associated with asthma in childhood and adult life, as well as with airway disease in experimental animals [6]. In pre-clinical studies, mice have been largely used to reproduce both asthma and obesity in humans [7]. Intranasal challenges with chicken egg ovalbumin (OVA) in animals previously immunized against this antigen evokes a large eosinophil infiltration into the airways within 24 to 48 h, which is accompanied by airway hyperresponsiveness, high levels of IL-4, IL-5, IL-13 and eotaxin—as well as mucus production, which partly reproduces the human asthma [8]. High-fat diet-induced obesity models also mimic the high eosinophilic airway inflammation of obese individuals [7,9,10].

Methylglyoxal (MGO) is one of the most important reactive dicarbonyl compounds derived from the glycolysis process [11,12]. High levels of MGO are found in serum and urine of pre-diabetic, diabetic and obese individuals, which has been associated with the worsening of these cardiometabolic conditions [13,14]. Intracellularly produced MGO can generate advanced glycation end products (AGEs) by non-enzymatic action glycation of free amino groups, which characterizes the post-translational protein modifications and its loss of function in cardiometabolic diseases [12]. AGEs promote their actions in different cell types and tissues by binding to its receptor RAGE, a transmembrane receptor and member of the immunoglobulin superfamily, further activating different intracellular signaling pathways that lead to increased production of pro-inflammatory and pro-oxidant mediators [15]. RAGE is highly expressed in lungs [16] and has been critically implicated in Th2-induced inflammatory responses and type-2 high asthma [17]. The increased production of reactive-oxygen species (ROS) by RAGE is also reported to mediate obesity-associated diseases [18]. Furthermore, prolonged exposure to MGO significantly increased the immunostaining and mRNA expression of RAGE in the mouse airways and exacerbated allergic and non-allergic inflammation [19,20]. Oral feeding with MGO significantly increased the mouse airway resistance and decreased maximal inspiratory flow, which was not observed in RAGE knockout mice [21].

Metformin is a classic oral anti-hyperglycemic agent used in the treatment of type 2 diabetes, acting mainly through the activation of the adenosine monophosphate-activated protein kinase (AMPK) pathway, thus reducing the insulin resistance and levels of glycated hemoglobin [22]. In mice fed with a high-fat diet for 30 weeks, metformin treatment significantly reduced the OVA-induced inflammation and remodeling [10,23]. Metformin can also act as a direct inhibitor/scavenger of MGO [24,25], thereby decreasing the concentrations of MGO in diabetic and obese individuals, which may explain at least in part the protective mechanisms of this anti-hyperglycemic agent [26]. Considering recent pre-clinical studies showing that chronic exposure of MGO to non-diabetic mice markedly potentiates airway inflammation by mechanisms associated with increased levels of RAGE and ROS [19,20], we thought that treatment with metformin could counteract the airway inflammation by scavenging MGO. Therefore, mice consuming 0.5% MGO for 12 weeks were pre-treated or not with metformin for 2 weeks, after which they were immunized and challenged with OVA. The airway inflammation and remodeling were then investigated.

## 2. Results

**Figure 1:**
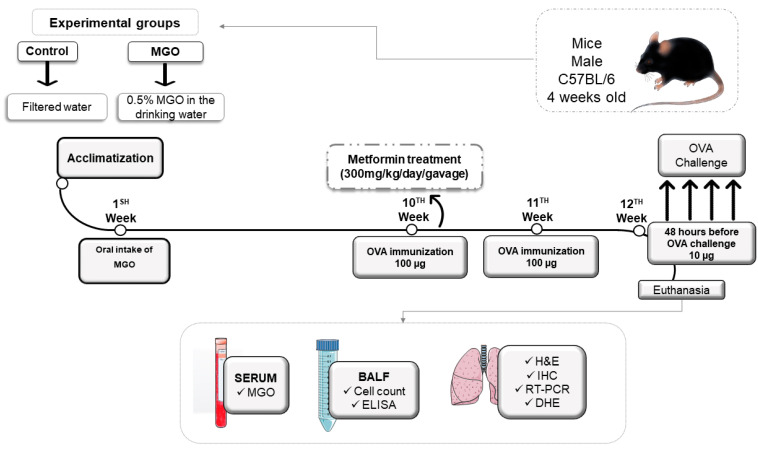
Experimental protocol of immunization and challenge with ovalbumin (OVA). Male C57BL/6 mice were treated or not with oral 0.5% methylglyoxal (MGO) for 12 weeks and then treated with metformin for the last 2 weeks. Some parts of the figure were drawn by using pictures from Servier Medical Art. Servier Medical Art by Servier is licensed Under a creative common attribution 3.0 Unported License (https://creativecommons.org/licenses/by/3.0/ accessed on 15–16 February 2023). BALF, bronchoalveolar lavage fluid; H&C, hematoxylin/eosin; IHC, immunohistochemistry; DHE, dihydroethidium.

Figure 1 illustrates the experimental protocols for airway immunization and challenge with OVA in mice under no exposure (control group) or exposed to MGO and treated or not with metformin. Briefly, mice were exposed to 0.5% MGO for 12 weeks and in the last 2 weeks received concomitant metformin (300 mg/kg, gavage), after which they were challenged with OVA (or instilled with phosphate-buffered saline; PBS).

### 2.1. Serum Levels and Lung Expression of MGO

**Figure 2:**
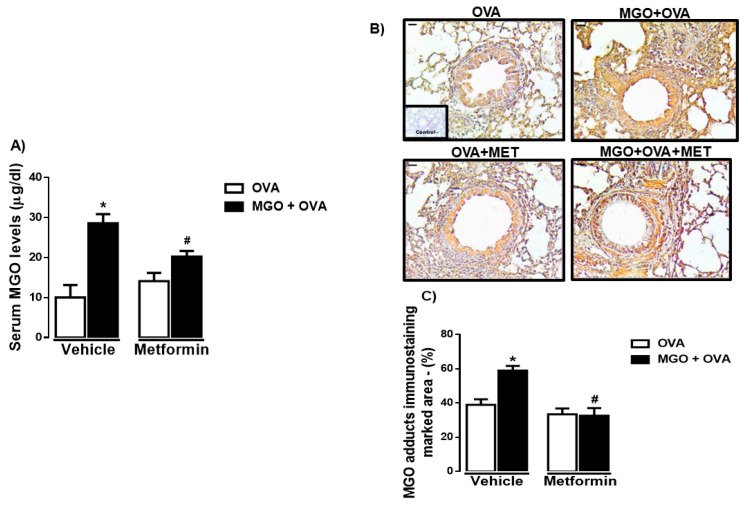
Levels of methylglyoxal (MGO) in serum (**A**) and immunohistochemical (IHC) staining for MGO in the airways of ovalbumin (OVA)-challenged mice (**B**,**C**). Mice were treated or not with 0.5% MGO for 12 weeks in the drinking water and metformin (MET; 300 mg/kg/day, gavage for 2 weeks), after which they were all intranasally challenged with OVA. Serum levels of MGO were measured by ELISA kit (**A**). Panel (**B**) shows the representative images of immunostaining expression for MGO in the peribronchiolar regions (staining in brown; bar = 100 μm and 200× objective). Panel **C** shows the MGO immunostaining quantification (marked area). Data in panels A and C are expressed as mean ± SEM for (*n* = 7–8). * *p* < 0.05 compared with respective vehicle group. ^#^ *p* < 0.05 compared with MGO + OVA in vehicle group.

Initially, we quantified the MGO levels in serum and airways, as performed by ELISA (Figure 2A) and immunohistochemistry for MGO adduct (Figure 2B,C), respectively. As expected, oral intake of MGO markedly elevated the serum levels of this dicarbonyl species, by about 2.8-fold (*p* < 0.05; Figure 2A). Treatment with metformin significantly reduced the serum MGO levels in these animals (Figure 2A). We also observed a high immunostaining for MGO adduct in the peribronchiolar regions of MGO-exposed mice, which was reduced by metformin treatment (Figure 2B,C). In animals under no exposure to MGO (control group), metformin affected neither the basal levels of MGO in serum nor the MGO adduct immunostaining in lung tissue (Figure 2A–C).

### 2.2. Analysis of Inflammatory Cell Migration in BALF and Lung Tissues

**Figure 3:**
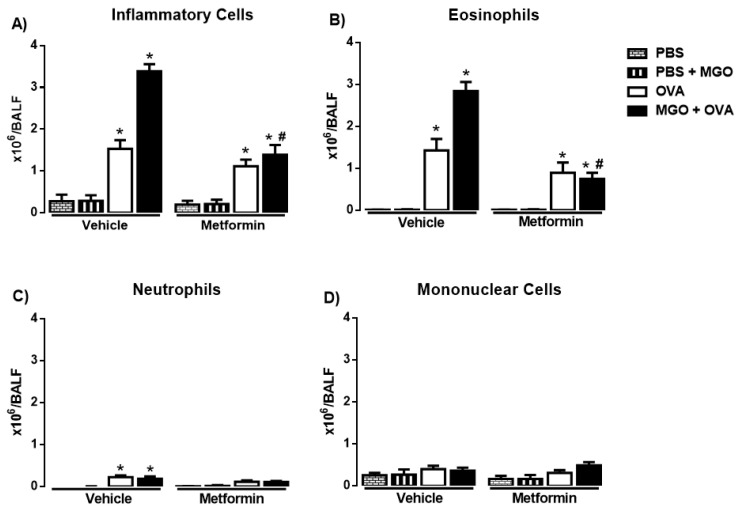
Number of total (**A**) and differential number of inflammatory cells (eosinophils, neutrophils, and mononuclear cells; (**B**–**D**)) in bronchoalveolar lavage fluid (**A**–**D**) in mice instilled with phosphate-buffered saline (PBS) or intranasally challenged with ovalbumin (OVA). Mice were treated or not with 0.5% methylglyoxal (MGO) for 12 weeks in the drinking water alone or combined with metformin (MET). Data are expressed as mean ± SEM for (*n* = 7–8). * *p* < 0.05 compared with the CT groups. ^#^ *p* < 0.05 compared with the MGO + OVA in vehicle groups.

The number of total inflammatory cells, eosinophils, neutrophils and mononuclear cells was evaluated in BALF in all groups (Figure 3A–D). Intranasal instillation with PBS (instead of OVA) resulted in virtually no eosinophil infiltration in BALF (total cells consisted essentially of mononuclear cells in PBS groups, *n* = 7–8; Figure 3A–D). As opposed, OVA-challenge in previously immunized mice markedly increased the number of total inflammatory cells and eosinophils in BALF at 48 h with the presence of few neutrophils (Figure 3A–C), confirming the efficacy of the OVA-sensitization and challenge procedure.

Compared with control group (animals under no exposure to MGO), the number of total inflammatory cells and eosinophils markedly increased (*p* < 0.05) in BALF of MGO-exposed mice, which was fully restored by metformin treatment (Figure 3A,B). Next, we examined the effects of metformin in the OVA-induced cell infiltration in mice exposed or not to MGO. In control group, metformin treatment had no significant effect on the number of total inflammatory cells and eosinophils, but in lungs of MGO-exposed mice, metformin suppressed the increased number of total cells and eosinophils in BALF (Figure 3A–D). The number of neutrophils and mononuclear cells in BALF remained unchanged in all groups (Figure 3C,D).

**Figure 4:**
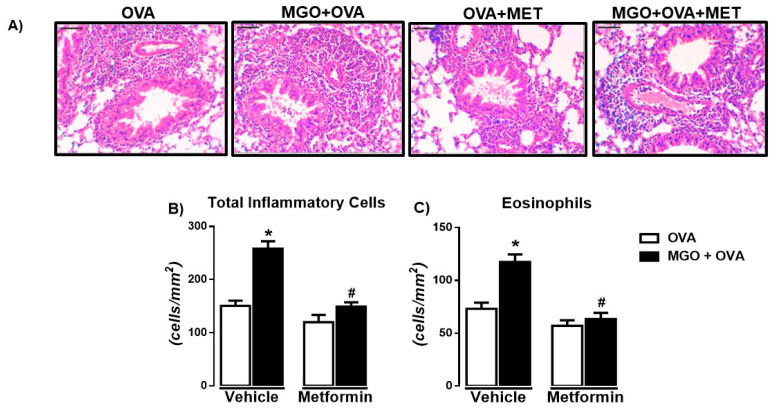
Representative images of hematoxylin and eosin (H&E) staining (**A**) and number of total inflammatory cells (**B**) and eosinophils (**C**) in lung tissue sections of ovalbumin (OVA)-challenged mice (bar = 200 μm; 200× objective). Mice were treated or not with 0.5% methylglyoxal (MGO) for 12 weeks in the drinking water alone or combined with metformin (MET). Data are expressed as mean ± SEM for (*n* = 7–8). * *p* < 0.05 compared with respective vehicle groups. ^#^ *p* < 0.05 compared with the MGO + OVA in vehicle groups.

Figure 4A–C shows the histopathology by H&E staining of the airways in all experimental groups. Like BALF, in OVA-challenge mice, a large infiltration of total inflammatory cells and eosinophils was observed, which was further increased by MGO exposure (*p* < 0.05). Treatment with metformin normalized the total cells and eosinophils in the lung sections of OVA-challenged mice exposed to MGO, without significantly affecting the cell infiltration in the lung sections of control group (Figure 4B,C). Very few numbers of neutrophils were visualized in the lung sections of OVA-challenged mice, exposed or not to MGO.

### 2.3. Levels of IL-4, IL-5, IL-13 and Eotaxin in BALF

**Figure 5:**
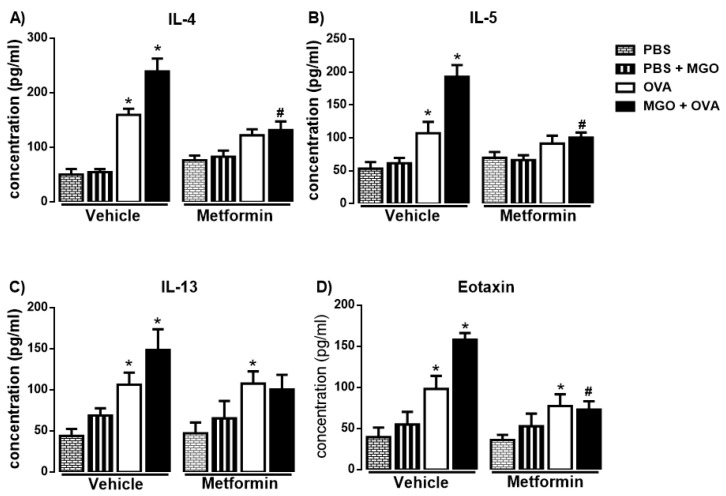
Levels of the Th2-cytokines IL-4, IL-5, IL-13 and eotaxin (**A**–**D**) in bronchoalveolar lavage fluid of mice instilled with phosphate-buffered saline (PBS) or intranasally challenged with ovalbumin (OVA). Mice were treated or not with 0.5% methylglyoxal (MGO) for 12 weeks in the drinking water alone or combined with metformin (MET; 300 mg/kg/day, gavage, 2 weeks). Data are expressed as mean ± SEM for (*n* = 7–8). * *p* < 0.05 compared with respective vehicle groups. ^#^ *p* < 0.05 compared with the MGO + OVA in vehicle groups.

In OVA-challenge mice, a significant increase in the levels of IL-4, IL-5, IL-13 and eotaxin was observed as compared with PBS group (Figure 5A–D; *p* < 0.05). The levels of IL-4, IL-5 and eotaxin were further elevated (*p* < 0.05) in BALF of MGO-exposed mice compared with control group, and that was suppressed by metformin treatment (Figure 5A,B,D). In MGO-exposed mice, no statistical differences between groups OVA groups treated or not with metformin were found for IL-13 levels (Figure 5C).

For the further protocols involving exposure to MGO and treatment with metformin, mice in all groups were always challenged with OVA.

### 2.4. Analysis of Airway Remodeling in Histological Sections of Lung Tissue

**Figure 6:**
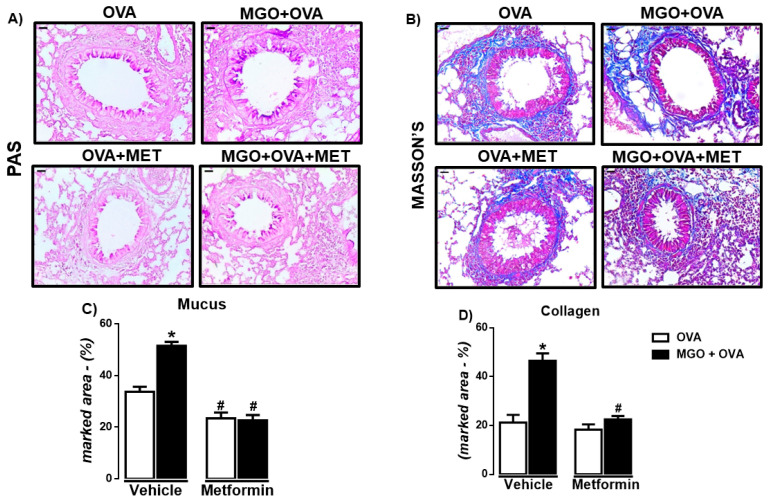
Representative images and quantification (%) of mucus production (**A**,**C**) and collagen deposition (**B**,**D**) in lung sections of ovalbumin (OVA)-challenged mice, according to periodic Acid-Schiff (PAS) and Masson’s trichrome, respectively (bar = 200 μm; 200× objective). Mice were treated or not with 0.5% methylglyoxal (MGO) for 12 weeks in the drinking water alone or combined with metformin (MET). Data are expressed as mean ± SEM for (*n* = 7–8). * *p* < 0.05 compared with respective vehicle groups. ^#^ *p* < 0.05 compared with the MGO + OVA in vehicle groups.

In lung tissue, we evaluated the airway remodeling by staining the histological sections with either Periodic Acid-Schiff (PAS) or Masson’s trichrome to identify mucus production and collagen deposition, respectively. The percentages of mucus (Figure 6A,C) and collagen (Figure 6B,D) were significantly higher in MGO when compared to control groups (*p* < 0.05), which were significantly decreased by metformin treatment. In control groups, metformin had no significant effect on collagen deposition (Figure 6D) but reduced by approximately 30% (*p* < 0.05) the mucus production (Figure 6C).

### 2.5. RAGE Levels in Lung Tissue and BALF

**Figure 7:**
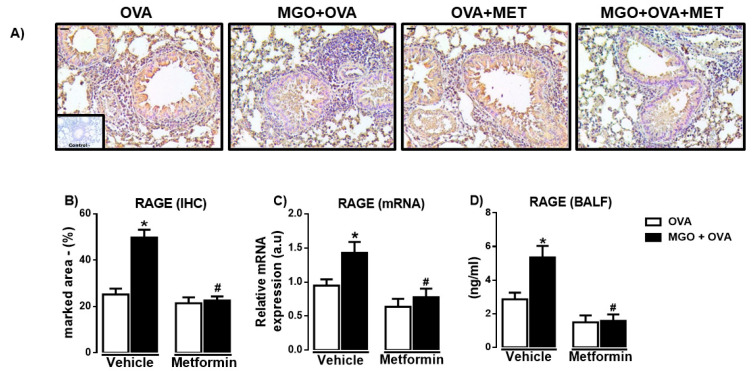
Expressions of the receptor for advanced glycation end products (RAGE) in the lung sections and bronchoalveolar lavage fluid (BALF) of ovalbumin (OVA)-challenged mice. Mice were treated or not with 0.5% methylglyoxal (MGO) for 12 weeks in the drinking water alone or combined with metformin (MET). Panels (**A**,**B**) show, respectively, representative images and percent quantification (% marked area) of RAGE by immunohistochemistry (IHC; brown staining) of the peribronchiolar regions (bar = 200 μm, 200× objective). Panel (**C**) shows the RAGE expression by RT-PCR in the lung homogenates, and panel (**D**) shows the levels in BALF. Data are expressed as mean ± SEM for (*n* = 7–8). * *p* < 0.05 compared with respective vehicle groups. ^#^ *p*< 0.05 compared with the MGO + OVA in vehicle groups.

We then evaluated the expression of RAGE in lung tissue through immunohistochemistry and qPCR techniques. Immunohistochemistry revealed the presence of RAGE in peribronchiolar regions in all groups, as detected in bronchial smooth muscle, epithelium and infiltrating inflammatory cells (Figure 7A). RAGE immunostaining was significantly higher in lung sections of MGO-exposed animals compared with the control group (Figure 7B). Likewise, the mRNA expression of RAGE in the lung tissue of MGO-exposed mice was higher than the control group (Figure 7C). Metformin treatment significantly reversed the high RAGE immunostaining and mRNA expression in lung tissue of MGO-exposed mice (*p* < 0.05), without affecting both parameters in the vehicle group (Figure 7A,B). We next evaluated the levels of RAGE in BALF (Figure 7D). Compared with the vehicle group, levels of RAGE in serum were also significantly higher in BALF of MGO-exposed mice (*p* < 0.05) and normalized by metformin treatment (Figure 7D).

### 2.6. ROS Levels and SOD Expression in Lung Tissue

**Figure 8:**
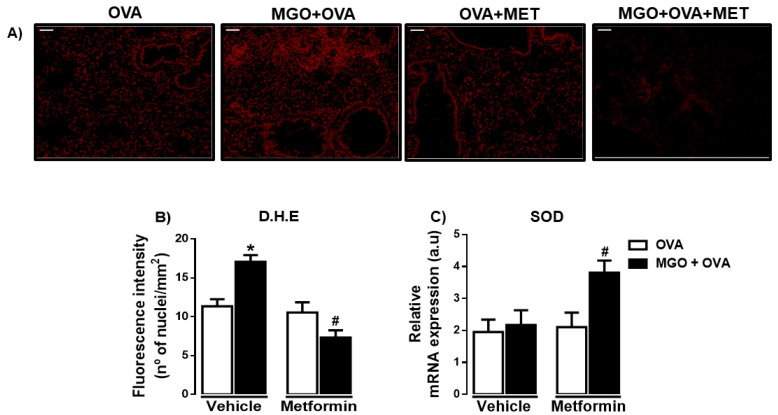
Representative images of the levels of reactive-oxygen species (ROS) (**A**) and quantification of dihydroethidium (DHE; bar = 200 μm, 200× objective) in lung tissues of ovalbumin (OVA)-challenged mice. Mice were treated or not with 0.5% methylglyoxal (MGO) for 12 weeks in the drinking water alone or combined with metformin (MET). Panel (**B**) Levels of ROS through dihydroethidium (DHE)-induced fluorescence in lung tissue and (**C**) shows the superoxide dismutase (SOD) expression by RT-PCR in the lungs. Data are expressed as mean ± SEM for (*n* = 7–8). * *p* < 0.05 compared with respective vehicle groups. ^#^ *p* < 0.05 compared with the MGO + OVA in vehicle groups.

We evaluated ROS levels in lung tissue by the immunofluorescence technique using DHE staining (Figure 8A,B). We observed that lung tissue of MGO-exposed mice exhibited higher levels of ROS compared with control group (*p* < 0.05; Figure 8B). Metformin treatment nearly abolished the increased ROS in MGO group, without significantly affecting the levels in control group. In addition, in MGO-exposed mice, metformin treatment significantly elevated the mRNA expression of SOD (*p* < 0.05) without affecting its expression in the other groups (Figure 8C).

## 3. Discussion

Long-term oral intake of MGO by healthy mice aggravates neutrophilic and eosinophilic airway inflammation by activating the AGE-RAGE pathway [19,20]. Metformin is reported to bind to MGO, acting as a direct scavenger of this dicarbonyl species [24,25,26,27]. We then sought to investigate if metformin treatment could reduce the MGO-mediated exacerbation of eosinophilic airway disease by inactivating MGO. Our data showed generally that metformin efficiently counteracted the effects of MGO, then restoring the inflammatory and remodeling markers to the levels of untreated mice.

In asthmatic individuals and experimental models of OVA-challenged mice it is well established that increased recruitment of eosinophils into airways worsens the prognosis of allergic airway disease [4]. Therefore, we evaluated the airway cell recruitment in both BALF and lung sections, as well as the levels of Th2 cytokines and eotaxin, which have been largely implicated in eosinophil functions [28]. IL-4 acts by increasing the regulation of adhesion molecules on endothelial cells, facilitating eosinophil infiltration into lung tissue, in a similar fashion with IL-13, while IL-5 acts by releasing signals through mast cells for mobilization and release from the bone marrow; eotaxin acts to increase the production and recruitment of eosinophils [29]. In OVA-challenged mice at 48 h (but not in mice instilled with PBS), we observed a marked infiltration of total inflammatory cells and eosinophils in the peribronchiolar regions. A negligible airway recruitment of neutrophils in BALF and lung sections of OVA-challenged mice was seen in all groups, which is consistent with murine models of allergic asthma [30]. Furthermore, the large eosinophil infiltration in BALF of OVA-challenged mice were accompanied by higher levels of Th2 cytokines (IL-4, IL-5 and IL-13) and eotaxin. The high eosinophil peribronchiolar recruitment and elevated levels of IL-4, IL-5 and eotaxin in OVA-challenged mice were further increased by MGO exposure, thus confirming our previous study [19]. Furthermore, two-week treatment with metformin counteracted all the potentiating effects of MGO on cell recruitment (BALF and lung section) and levels of IL-4, IL-5 and eotaxin (BALF). Indeed, the oral exposure to MGO significantly elevated the serum concentration of this dicarbonyl species in the OVA-challenged mice, and that was also reduced by metformin treatment. This is consistent with previous studies demonstrating that metformin treatment reduces the plasma MGO levels in patients with type 2 diabetes [26]. Likewise, in lung sections of OVA-challenged mice, exposure to MGO increased the immunostaining of MGO adduct that was also normalized by metformin, strongly suggesting that metformin itself inactivates the molecule MGO, thus preventing its deleterious action in the lungs. Treatment with metformin alone (in the absence of MGO exposure) had a slight tendency to reduce the OVA-induced eosinophilic airway inflammation (despite no statistical differences being found), which may indicate its capacity to inactivate endogenously produced MGO.

Structural changes in the lung of asthmatic patients and animals include epithelial and subepithelial thickening due to collagen deposition [31], and excessive collagen deposition can worsen obesity-related asthma [32]. Collagen is susceptible to glycation by MGO, and glycated collagen may exhibit a greater stiffness in affected tissues [33]. However, no study exists evaluating the involvement of MGO in airway collagen deposition. Another important factor linked to the pathophysiology of asthma is the excessive production of mucus in the airways [34]. In cultured human nasal epithelial cells, MGO was shown to dose-dependently increase the mucus secretion [35], whereas in a mouse model of neutrophilic airway inflammation, RAGE was associated with mucus hypersecretion [36]. In our study, MGO exposure markedly increased both airway collagen deposition and mucus production. Metformin treatment fully counteracted the effects of MGO exposure on collagen deposition and mucus secretion in the mouse lung of OVA-challenged mice.

Endogenous MGO as generated by the auto-oxidation of glucose acts as a main precursor of AGEs, which in turn exert numerous deleterious functions mainly via binding to RAGE receptor [37,38]. RAGE is a multifunctional receptor and member of the immunoglobulin superfamily of cell surface receptors that amplifies inflammatory and immunological processes [39,40]. In the airways, RAGE serves as a critical factor in the initiation of Th2-mediated airway inflammation [16,17] and epithelial dysfunction [41]. Moreover, many of the RAGE actions are due to activation of NADPH oxidase, which leads to excess formation of ROS, thus contributing to generate a pro-oxidant environment [42,43]. Elevated production of ROS is increased in the lung tissue of asthmatic patients and OVA-induced mice [19,44,45]. Antioxidant enzymes such as superoxide dismutase (SOD) play a crucial role in different pathophysiological processes such as asthma and diabetes [46]. We then studied RAGE expressions by both immunohistochemistry and Real-Time RT-PCR gene expression in lung tissue, as well as by measuring the RAGE levels in BALF (ELISA assays). We also evaluated the ROS levels and SOD mRNA expression in the lung sections. We observed that MGO exposure significantly elevated the RAGE immunostaining and mRNA expression in lung tissues and RAGE levels in BALF of OVA-challenged mice, all of which were markedly reduced by metformin treatment. The elevated ROS production by MGO exposure was also normalized by metformin and that was accompanied by a high SOD expression in lung tissue, which is consistent with a previous study showing in human renal proximal tubular cells that metformin blocked the AGEs-RAGE-ROS-induced cell injury in vitro [47].

Clinical studies carried out in both asthmatic and diabetic individuals show that metformin users exhibit lower risks of asthma-related exacerbation, hospitalization and/or emergency visits compared with non-users [48,49], despite the fact that the efficacy of metformin as an adjuvant therapy in asthmatic patients has not been conclusive. In the present study, non-diabetic healthy mice exposed to 0.5% MGO for 12 weeks (in association or not with metformin) reveal no alterations in glycemic parameters such as fasting glucose levels and insulin sensitivity [19,50]. This suggests that mechanisms other than suppression of hepatic glucose production explain the protective effects of metformin in OVA-induced murine airway inflammation, which could reflect indeed its MGO scavenging property. The dose of metformin used in mice (300 g/kg for 2 weeks) was much larger than that prescribed for patients with type 2 patients (usually ranging from 500 to 2000 mg daily); therefore, whether this MGO scavenging property of metformin would be better achieved at higher non-conventional doses of this anti-hyperglycemic requires additional studies.

## 4. Materials and Methods

### 4.1. Animals

An amount of 4-week-old male C57BL/6 mice obtained from the Multidisciplinary Center for Biological Investigation (CEMIB) at the University of Campinas (UNICAMP) were housed in cages (3 or 4 per cage) located in ventilated cage shelters with constant humidity (55 ± 5%) and temperature (23 ± 1 °C) under a 12 h light-dark cycle. Animals received standard food *ad libitum*. Animal procedures and experimental protocols are in accordance and were approved by the Ethics Committee in Animal Use (CEUA-UNICAMP; protocol number 6081-1/2022, and followed the Brazilian Guidelines for the Production, Maintenance and Use of Animals for Teaching or Research of the National Council of Control in Animal Experimentation (CONCEA).

### 4.2. MGO and Metformin Treatments

Mice received 0.5% MGO (Sigma Aldrich, MI, USA) in drinking water for 12 weeks, according to previous studies [50,51]. Metformin was administered daily by gavage at 300 mg/kg [21] in the final 2 weeks of MGO treatment. Mice were immunized and challenged with ovalbumin (OVA), as detailed below. Thus, most of our experimental protocols resulted in 4 experimental groups of 7 to 8 mice each, totaling 60 mice, as follows: (i) Control + Vehicle + OVA, (ii) Control + Metformin + OVA, (iii) MGO + Vehicle + OVA, and (iv) MGO + Metformin + OVA. In some protocols, mice intranasally instilled with PBS (50 µL) instead of OVA served as controls of the OVA-challenged mice.

### 4.3. Induction of Airway Inflammation: Immunization Procedure and OVA Challenge

Mice were actively immunized with a subcutaneous injection (0.4 mL) of 100 µg OVA (grade V; Sigma-Aldrich Co., St Louis, MO, USA) mixed with 1.6 mg of Al(OH)3 in 0.9% NaCl on days 0 and 7. On days 14 and 15, immunized mice were challenged intranasally with OVA (10 µg/50 µL) twice daily, resulting in 4 challenges—that is, the first challenge occurred at the time 0 and the second challenge 6 hours later; on the second day, the third challenge occurred at time zero and the fourth challenge at 6 hours later. Forty-eight hours after the first challenge, the mice were euthanized with isoflurane and exsanguinated. Euthanasia was performed by isoflurane overdose, in which animals were exposed to a concentration greater than 5% for up to 1 minute after cessation of breathing and cervical displacement was performed to confirm euthanasia. Bronchoalveolar lavage fluid was performed, and the lungs were collected.

### 4.4. Bronchoalveolar Lavage Fluid (BALF)

The animal trachea was exposed through an incision, and then cannulated with a polyethylene tube connected to a syringe. The lungs were washed 5 times each with 300 µL of PBS through the tracheal cannula. The fluid recovered after each wash was combined and centrifuged at 500 g for 10 min at 4 °C, and BALF supernatant was stored at −80 °C. The cellularity that forms the pellet was resuspended in 200 µL of PBS. Total (Neubauer) and differential cell counts were made with (Diff-Quick stain). A minimum of 300 cells were counted and classified as eosinophils, neutrophils and mononuclear cells based on normal morphological criteria.

### 4.5. Lung Morphometric and Immunohistochemistry Analysis

The lungs were collected and washed with 10 mL of PBS and subsequently immersed in 10% phosphate-buffered formalin for 24 h and kept in 70% ethanol until incorporation into paraffin. Tissues were sliced (5-μm sections) and stained with hematoxylin/eosin (H&E), periodic acid-Schiff, or Masson’s trichrome to use as markers of mucus secretion and subepithelial fibrosis, respectively, by light microscopy examination. Morphometric analysis was performed using a Leica DM 5000B digital camera. All quantitative analysis on lung tissue was performed by two experimenters, one of whom was blinded. The lung images were evaluated using detected by the ImageJ Software (ImageJ, U. S. National Institutes of Health, Bethesda, MD, USA, https://imagej.nih.gov/ij/, 1997–2023 (accessed on 28 September 2022). For each different color, the area of positivity was measured in mm^2^ for five bronchioles per slide.

For immunohistochemistry, the lung tissue sections were dewaxed and rehydrated in xylol and serial ethanol solutions. Antigenic retrieval was performed using 10 mM sodium citrate buffer through the heating method and endogenous peroxidase was blocked in 0.3% hydrogen peroxide. After being blocked by 5% bovine serum albumin (BSA), the lung slides containing the lung sections were incubated overnight at 4 °C with a mouse monoclonal anti-RAGE primary anti-antibody (1:1000; cat. no. ab216329, Abcam, Cambridge, UK) or anti-MGO adduct (1:500; cat. no. ab243074, Abcam, Cambridge). All sections were labeled with the secondary antibody, using the Mouse ExtrAvidin-peroxidase staining kit (1:1000; Cat. no. EXTRA2, Sigma St Louis, MO, USA) and the stained areas were detected using 3.3′ diaminobenzidine solution (DAB; Cat. no. D4293, Sigma St Louis, MO, USA). Lung imaging was performed using a Leica DM 5000B digital camera.

### 4.6. Measurements of Levels of Cytokines, RAGE and MGO

Levels of IL-4, IL-5, IL-13 and eotaxin in BALF were measured using commercially available Duo Set ELISA kits (R&D, Minneapolis, MN, USA). RAGE levels in BALF were measured using a Mouse RAGE ELISA kit (Cat. no. ab100738, Abcam, Cambridge, UK). Serum levels of MGO were measured using ELISA competitive kit for OxiSelect™ Methylglyoxal (Catalog No. STA-811, Cell Biolabs, San Diego, CA, USA). Plates were read in a Hybrid Multi-Mode Microplate Reader Synergy™ H1 (BioTek Instruments, Winooski, VT, USA).

### 4.7. Real-Time RT-PCR Gene Expression for RAGE and Superoxide Dismutase (SOD)

The total RNA was extracted from homogenized lungs using TRIzol^®^ reagent (Invitrogen, Ann Arbor, MI, USA) and DNase treated RNA samples were then transcribed with High-Capacity Reverse Transcription Kit^®^ (Applied Biosystems, Carlsbad, CA, USA). cDNA samples concentrations were quantified using a spectrophotometer (Nanodrop Lite^®^, Thermo Scientific, Waltham, MA, USA). Synthetic oligonucleotide primers (Table 1) were obtained from Integrated DNA Technologies (Coralville, IA, USA) and Qiagen (Hilden, Germany). The reactions were performed with 10 ng cDNA, 6 µL SYBR Green Master Mix^®^ (Life Technologies, Carlsbad, CA, USA) and the optimal primer concentration in a total volume of 10 μL. Real-time PCR was performed in the equipment Step One-Plus^®^ Real Time PCR System (Applied Biosystems). Threshold cycle (Ct) was defined as the point at which the fluorescence rises appreciably above the background fluorescence. To determine the specificity of the amplification, the melting curve analysis of the PCR products was performed to ensure that only one fragment was amplified. The 2^−ΔΔCt^ method was utilized to analyze the results, which were expressed by the difference between Ct values of chosen genes and the housekeeping gene, the 18S ribosomal RNA (18S rRNA), in which the signal strength did not differ between the control and MGO groups (Ct: 8.5 ± 0.3 and 8.6 ± 0.4, respectively).

### 4.8. Quantification of Reactive-Oxygen Species (ROS) in the Lungs

The collected lungs were submitted to a Tissue-Tek OCT (Sakura, CA, USA) freezing process and stored at −80 °C. Using a cryostat, 10-μm lung sections from each animal were obtained. The lung sections were then placed on glass slides and maintained at 37 °C for 20 min, after which they were incubated with dihydroethidium (DHE, 2 μM) diluted in phosphate buffer for 30 min at 37 °C in a humid chamber. The sections were observed with a fluorescence microscope (Eclipse 80i, Nikon, Japan) equipped with a camera (DS-U3, Nikon, Japan) using a rhodamine filter. At 200× magnification, 10 different images were randomly acquired from each section. The intensity of fluorescence was determined using the ImageJ Software (ImageJ, U. S. National Institutes of Health, Bethesda, MD, USA, https://imagej.nih.gov/ij/, 1997–2023 (accessed on 2 October 2022).

### 4.9. Statistical Analysis

Data were expressed as means ± SEM. The program GraphPad version 6.0 software was used for statistical analysis. Statistically significant differences were determined using one-way analysis of variance (ANOVA) for multiple comparisons followed by Tukey test. A value of *p* < 0.05 was accepted as significant.

## 5. Conclusions

The potentiation of OVA-induced airway eosinophilic inflammation and remodeling by MGO exposure is counteracted by metformin treatment that acts by inactivating MGO, thus suppressing RAGE-ROS activation in lung tissue, in addition to exerting an antioxidant action in the lungs. Metformin may be an adjuvant pharmacological therapy to improve asthma in hyperglycemic/obese individuals with high levels of MGO.

## Figures and Tables

**Table 1:** Primer sequences used for real-time PCR amplifications.

IDT Integrated DNA Technologies		
**Gene**	**Forward**	**Reverse**
RAGE	5′-CTGAACTCACAGCCAGTGTCCC-3′	5′-CCCTGACTCGGAGTT-3′
SOD	5′-CAGCATGGGTTCCACGTCCA-3′	5′-CACATTGGCCACACCGTCCT-3′
18S rRNA	5′-GTAACCCGTTGAACCCCATT-3′	5′-CCAT CCAATCGGTAGTAGCG-3′

Abbreviations: RAGE, receptor for advanced glycation end products; SOD, superoxide dismutase; 18S rRNA, 18S ribosomal RNA.

## Data Availability

The data underlying this article will be shared on reasonable request to the corresponding author.
